# Ginsenoside Metabolite Compound K Promotes Recovery of Dextran Sulfate Sodium-Induced Colitis and Inhibits Inflammatory Responses by Suppressing NF-κB Activation

**DOI:** 10.1371/journal.pone.0087810

**Published:** 2014-02-04

**Authors:** Juan Li, Wei Zhong, Weiwei Wang, Shaoping Hu, Jiahui Yuan, Bing Zhang, Tianhui Hu, Gang Song

**Affiliations:** 1 Cancer Research Center, Medical College of Xiamen University, Xiamen, China; 2 Department of Basic Medicine, Medical College of Xiamen University, Xiamen, China; Université Paris Descartes, France

## Abstract

Phytogenic compounds with anti-oxidant and anti-inflammatory properties, such as ginsenoside metabolite compound K (CK) or berberine (BBR), are currently discussed as promising complementary agents in the prevention and treatment of cancer and inflammation. The latest study showed that ginsenoside Rb1 and its metabolites could inhibit TNBS-induced colitis injury. However, the functional mechanisms of anti-inflammation effects of ginsenoside, particularly its metabolite CK are still not clear. Here, using dextran sulfate sodium (DSS)-induced colitis in mice, clinical parameters, intestinal integrity, pro-inflammatory cytokines production, and signaling pathways in colonic tissues were determined. In mild and sever colitis mice, CK and BBR (as a positive agent) alleviated colitis histopathology injury, ameliorated myeloperoxidase (MPO) activity, reduced pro-inflammatory cytokines production, such as, IL-6, IL-1β, TNF-α, and increased anti-inflammatory cytokine IL-10 production in both mice colon tissues and blood. Nevertheless, the results revealed that CK and BBR inhibited NF-κB p65 nuclear translocation, downregulated p-IκBα and upregulated IκBα, indicating that CK, as well as BBR, suppressed the activation of the NF-κB pathway in the progression of colitis with immunofluorescence, immunohistochemical and western blotting analysis. Furthermore, CK inhibited pro-inflammatory cytokines production in LPS-activated macrophages *via* down-regulation of NF-κB signaling pathway. Taken together, our results not only reveal that CK promotes the recovery of the progression of colitis and inhibits the inflammatory responses by suppressing NF-κB activation, but also suggest that CK downregulates intestinal inflammation through regulating the activation of macrophages and pro-inflammatory cytokines production.

## Introduction

Inflammatory bowel disease (IBD), which includes ulcerative colitis and Crohn’s disease, is associated with chronic, relapsing inflammation of the intestinal tract. Evidence from immunological, microbiological, and genetic studies suggest that IBD results from dysregulation of the mucosal immune system leading to excessive immunological responses to intestinal microflora, or changes in the composition of intestinal microflora and/or deranged epithelial barrier function that elicits pathological responses from the normal mucosal immune system in genetically susceptible hosts [1]–[5]. Nevertheless, acute intestinal inflammation is usually followed by physiologic healing of the damaged tissue and restoration of the normal structure and function of the intestine [6]. If the innate physiologic healing doesn’t work, acute inflammation can develop and character by continues events of injury, which associated with the innate immune system responses [7]. Moreover, in IBD and experimental model of autoimmune colitis in mice, when the innate immune responses are initiated by inflammation lesions, innate immune cells such as macrophage and intestinal epithelium cells will secret several cytokines and chemokines, including IL-6, IL-1β, TNF-α, which trigger the adaptive immune system including T and B cell-mediated responses [6]–[8]. Strikingly, unrestrained reaction may exaggerate inflammatory response and lead to intestinal damage [9]. Therefore, appropriate regulation of the innate immune reaction is very important to the severity of inflammation, so a clear understanding of the mechanism of the development and progression of IBD and colitis is crucial for researching new effective drugs on its therapy.

**Figure 1:**
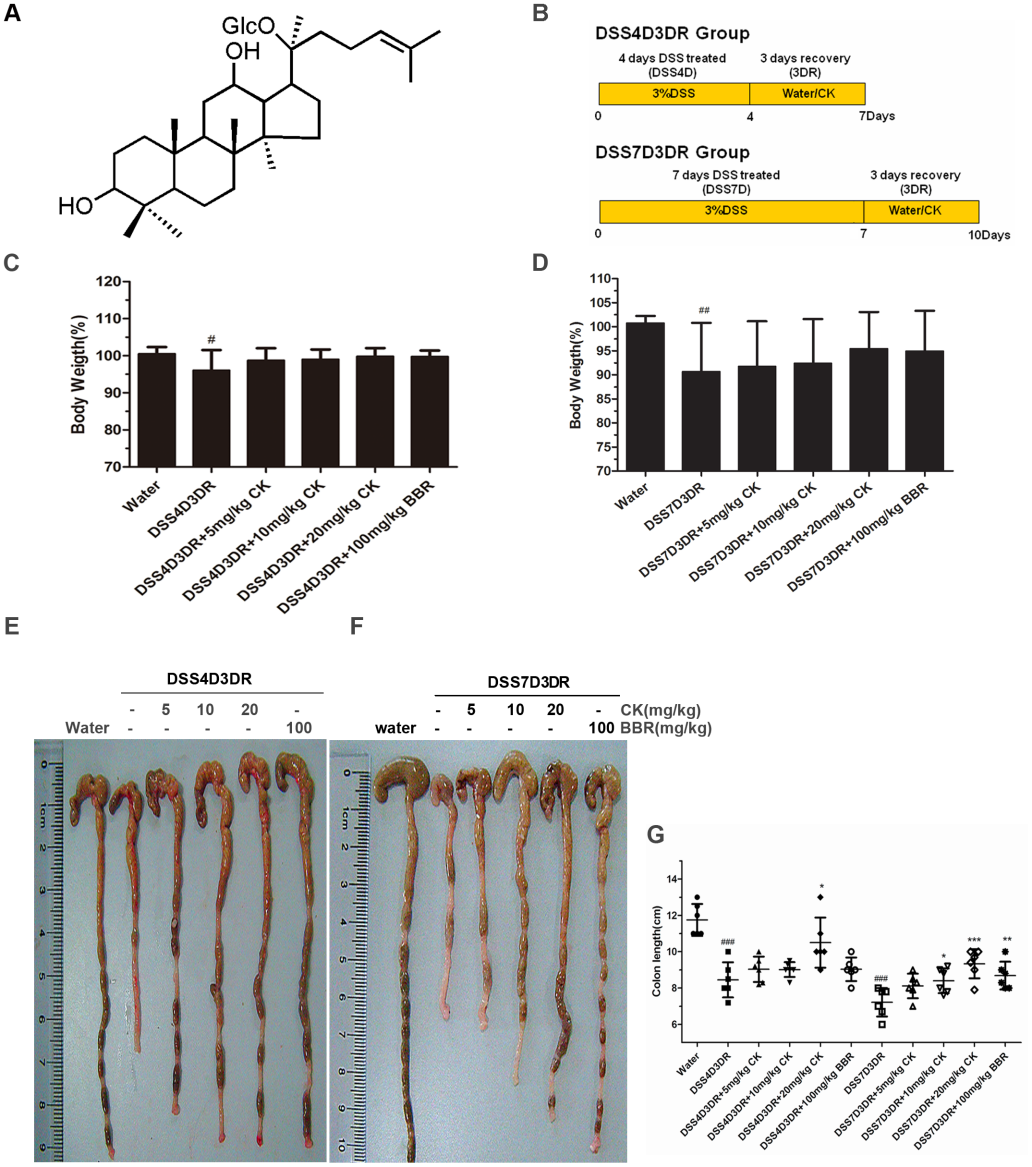
Anti-inflammatory influences of CK in DSS-induced colitis in mice. (A) The chemical structure of CK. The structure was elucidated to be 20-O-(β-D-glucopyranosyl)-20(S)-protopanaxadiol. (B) DSS-induced intestinal injury and inflammation in mice. Mice were administered 3% DSS in drinking water to induce colitis. CK and BBR were intraperitoneal injection for mild colitis group (4-day DSS treatment and 3-day recovery) and severe colitis group (7-day DSS treatment and 3-day recovery). Control mice received water alone. (C) The body weight of mild colitis mice were measured every day and presented as percentage of original body weight. Mild colitis mice: 4-day DSS treatment and 3-day recovery, from day 1 to day 7. (D) The body weight of mice were measured every day and presented as percentage of original body weight. Severe colitis mice: 7-day DSS treatment and 3-day recovery, from day 1 to day 10. (E) The colon morphology of mild colitis group. (F) The colon morphology of severe colitis group. (G) The lengths of colon were measured when mice were euthanized. n = 6. # *p<*0.05 compared with the control group. ## *p<*0.01, ### *p<*0.001 compared with the control group. *p compared with DSS4D3DR or DSS7D3DR group. *p<0.05, **p<0.01, ***p<0.001.

Nowadays, lots of Chinese herbal medicines, such as ginsenosides and berberine (BBR), have shown various beneficial therapy effects, including cancers and inflammations [10]–[17]. Compound K (20-O-beta-D-glucopyranosyl-20(S)-protopanaxadiol (structure shown in Fig. 1A) is the main metabolite of the protopanaxadiol type of ginsenoside produced by intestinal bacteria after oral administration of ginseng extracts and is speculated to be the major form of protopanaxadiol saponin absorbed from the intestine [10], [11]. Several studies have shown that CK possesses various chemopreventive and chemotherapeutic activities, including attenuation of hepatic lipid accumulation [11], antigenotoxicity and anticlastogenicity [12], reverse of multidrug resistance [13], and antitumor action [14], [15]. Our previous work have confirmed the effects of CK on suppression of hepatocellular carcinoma cells survival and its mechanisms of anti-metastatic growth associated with NF-κB p65 nuclear export and the inhibition of MMP2/9 expression [13]. Moreover, CK can also inhibitgrowth of gastric carcinoma and colorectal cancer (CRC) via regulating different pathways [10], [14], [15]. A latest study indicated that ginsenoside Rb1 and its metabolites inhibited TNBS-induced colitis injury, and reduced pro-inflammatory cytokines production in colon tissues [12]. However, the functional mechanisms of anti-inflammation effects of ginsenoside, especially its metabolites, are still not clear. The purpose of this work was to determine the inhibitory effects of CK on the progression of DSS-induced colitis, and to explore its efficiency mechanism. In this study, 4 days DSS-induced mild colitis mice and 7 days DSS-induced sever colitis mice *in vivo* and RAW 264.7 macrophages *in vitro* were used.

BBR (C20H19NO5) is an isoquinoline alkaloid isolated from coptidis rhizoma and cortex phellodendri [16], [17]. Previous studies have reported that BBR have therapeutic effects on DSS-induced colitis, including amelioration of colon injury, decrease of pro-inflammatory cytokines production and inhibition of the NF-κB pathway activation in colon tissues. BBR alsopromoted the peritoneal macrophage apoptosis, and regulated inflammatory responses by decreasing pro-inflammatory cytokines production in colonic macrophages and epithelial cells [1]. Thus, BBR was used as a positive agent for treating colitis mice here.

Our data showed that both in mild colitis groups and sever colitis groups, CK potently alleviated the colon histomorphology injury, promoted the recovery of the colitis and reduced pro-inflammatory cytokines production in a concentration-dependent manner. In addition, immunofluorescence, immunohistochemical and western blotting analysis indicated that CK inhibited colon tissues’ NF-κB pathway activation in a concentration-dependent manner. As in the inflammation function cycle, the macrophages stimulated and secreted cytokines and then regulated the innate inflammatory systems [18], [19]. According to what we have found, and to verify the functional mechanisms of CK in the intestinal disorder, LPS-stimulated macrophages (RAW 264.7 cells) treated with or without CK or BBR for 10 h, and then cytokines production and signaling pathways were determined *in vitro*. The results demonstrated that CK reduced the pro-inflammatory cytokines levels in LPS-stimulated macrophages by suppressing NF-κB activation in a dose-dependent manner.

Based on the existing data concerning CK, our present study assessed anti-colitis activity of CK with different inflammatory indexes and determined that CK promotes the recovery of colitis in mice associated with regulation of innate immune cells, such as macrophages, by suppressing NF-κB pathway activation. These finding may provide new point on CK treated intestinal inflammatory disorder.

## Materials and Methods

### Mice and Ethics Statement

Male BALB/C mice, weighing 180–200 g were used between periods of 3–5 weeks. All experimental mice were maintained under SPF conditions and raised under standard conditions (12-hour day-night rhythm) in Xiamen University Laboratory Animal Center. All animal procedures were approved by the Animal Care and Use Committee of Xiamen University (license No: SYXK [Min] 2008-0003, issued on May 6, 2008).

### Reagents and Cell Culture

Dextran Sulfate Sodium (molecular weight 36–50 kDa, MP Biomedical, Solon, OH), CK (CK was prepared and identified as in our previous publication (Fig. 1A, >98% pure as determined by HPLC) and BBR (Sigma-Aldrich, St. Louis, MO) were prepared and identified as in our previous publications [1], [13]. Antibodies NF-κB (p65) and IκBa were provided by Santa Cruz. p-IκBα purchased from Cell Signaling. The mouse RAW 264.7 macrophage cell line was obtained from the Institute of Biochemistry and Cell Biology, Chinese Academy of Sciences in Shanghai, and was cultured in DMEM medium containing 10% FBS, 1% glutamine, 100,000 IU/l penicillin, and 100 mg/l streptomycin at 37°C with 5% CO_2_ and 95% air.

### DSS-induced Colitis in Mice and CK Treatment

The curative effects of CK were investigated in the male BALB/C mice. The mice were divided into twelve groups, those are, normal control group and mild DSS-induced colitis groups (DSS four days) and sever colitis groups (DSS seven days) treated with or without CK (5, 10 or 20 mg/kg) or BBR (100 mg/kg). Colitis were induced by the orally administered of 3% (w/v) DSS solution double distilled water every day. The normal control group was treated with double distilled water alone. CK (5, 10 or 20 mg/kg) or BBR (100 mg/kg) was intraperitoneal injection into mice every day after DSS treatment to the day before sacrifice for 3 days. To assess the extent to which colitis was induced, the mice were examined daily for body weight, stool consistency, and blood in the stool.

### Analysis of Colon Injury

Paraffin-embedded tissue sections of Swiss-rolled whole colon (rolled from the distal end to the proximal end) were stained with hematoxylin and eosin for light microscopic examination to assess colon injury and inflammation. Samples from the entire colon were examined by a pathologist blinded to treatment conditions. A modified combined scoring system including degree of inflammation (scale of 0–3) and crypt damage (0–4), percentage of area involved by inflammation (0–4) and crypt damage (0–4), and depth of inflammation (0–3) was applied for assessing colitis induced by DSS. The total score is 0 (normal) to 18 (severe colitis).

### Blood Samples and Mice Tissues

7 days and 10 days after CK injection, the mice were scarified, a 1 ml sample of orbital blood were collected and the abdomens were opened along the median line, and then was rapidly excised, the colon gently with ice-cold PBS, placed on ice, and opened longitudinally. The colon was incised, and the fecal contents were washed out gently with of PBS. The blood samples were injected into dry test tubes and separated by centrifugation, and the serum was stored at −80°C until use. And then the colon was embed into the dry tubes and stored at −80°C until use.

### Immunofluorescence Staining

The colon tissues were fixed in 10% formaldehyde and embedded in paraffin. Tissue sections were permeabilized and blocked in PBS containing 0.3% Triton X-100 (Sigma-Aldrich, Milwaukee, WI) and 10% goat serum, followed by staining with primary antibodies for p65 (1∶300) over night at 4°C and an Cy3 secondary antibody (1∶200). p65 proteins were then detected and immunolocalized using Vectashield Mounting Medium containing DAPI. All the sections observed using fluorescence microscopy. Cultured cells immunofluorescence staining was performed as previously described [14]. Image-pro plus software was used to quantify the staining density. Based on the principles of equidistance, we randomly chose five views of distal end of colon in each section using 20X image, quantified the IOD and then averaged IOD of each section. Every group has prepared 10 sections. The averaged IOD of each group was compared by Student’s t-test.

### Immunohistochemical Staining

The colon tissues sections immunohistochemical staining was performed as previously described [13], [14]. As a negative control, the primary antibody was replaced with normal mouse IgG. The method of quantify the staining density is the same as immunofluorescence staining analysis.

### Detection of MPO Activity in Colon Tissues and Blood Plasma

This performs of MPO activity as described by the manufacturer (Nanjing Jiancheng Bioengineering Institute, China). Briefly, the colon was weighted, fixed with 19 fold phosphate buffer two, homogenated. The blood plasma mixed with buffer two in 1∶1. And then, both of them 0.9 mL homogenate added 0.1 mL buffer three, homogenated and putted in the 37°C water bath for 15 min. According to the protocol, buffer four, TMB Substrate and buffer six were fixed in ration, mixed and putted in the 60°C water bath for 10 min. The levels of MPO of colon tissues and blood plasma were be detected in 460 nm absorbance values.

### Enzyme-linked Immunosorbent Assay

Weighing the same weight of colon organization, fixed with ice-cold PBS (1∶9), homogenated, and centrifugalized (10000 rpm/min, 10 min). The supernatant were reserved. The macrophage RAW 264.7 cells were treated with lipopolysaccharide (LPS) (1 µg/ml) for 10 h in the presence or absence of different dose of CK (10 µM, 20 µM, 50 µM) and BBR (50 µM). After CK or BBR treatment, the supernatants of RAW 264.7 cells were collected and centrifugalized (1000 rpm/min, 5 min). The production of IL-1β, IL-6, TNF-α and IL-10 in colon tissues supernatants, blood serum and RAW 264.7 cell supernatants were determined in duplicate using ELISA kits (R&D System Europe Ltd., UK) as described by the manufacturer.

### Cytosolic, Nuclear Protein Isolation

The cytosolic, nuclear protein of colon tissues and RAW 264.7 cells fractions were isolated according to our previously reported procedure [13], [14]. Briefly, tissues and cells were scraped in ice-cold homogenization buffer. And then re-suspended in 5 volumes of ice-cold extract buffer A and were homogenized. The homogenates were putted in ice for 15 min, and then added buffer B and centrifuged at 16000 g for 5 min to obtain the cytoplasm pellets. The supernatants were further fixed buffer C and putted in ice for 40 min. The supernatants centrifuged at 160000 g for 1 h to collect the supernatants (the nuclear fraction).

### Western Blotting Analysis

Western blotting was performed as in our previous publications [13], [14]. Protein assay kit for protein quantity analysis was purchased from Bio-Rad (Hercules, CA). The enhanced chemiluminescence (ECL) detection system was purchased from Arlington Heights, IL. IamgeJ2X software was used to analyze the relative density of each band.

### Statistical Analysis

All values are expressed as the means ± S.D. for at least three separate determinations for each group. Statistical significance for multiple comparisons in each study was determined by one-way ANOVA followed by Newman-Keuls analysis using Prism 5.0 (GraphPad Software). Statistical analysis was performed by Student’s t-test.

## Results

### Anti-inflammatory Effects of CK in DSS-induced Colitis in Mice

The DSS mouse model of acute colitis is well-characterized by increased epithelial injury in previous work [2]–[5], and it has been proved that BBR has influence on promoting the recovery of DSS-treated colitis [1]. To confirm the efficiency of anti-inflammatory effects of CK and explore its functional mechanism, mice were treated with DSS for 4 days to induce mild colitis, and then CK was administered *via* intraperitoneal injection for the following 3 days recovery (Fig. 1B, upper panel). To induced severe colitis, mice were treated with DSS for 7 days, and CK was given to mice for the following 3 days recovery periods (Fig. 1B, lower panel). The mice which were just given water were used as control. In the mild colitis group, the mice body weight reached 96.08±5.41% of their original body weight at the average (*p<*0.05), but the CK or BBR treatment groups have no statistical significance (Fig. 1C). However, in the severe colitis group, the body weight began to lose after 5 days until the day end of the DSS treatment. When given CK or BBR, the body weight began to recover from day 8 to day 10. CK (20 mg/kg) and BBR (100 mg/kg) significantly improved body weight recovery from day 8 to day 10 (Fig. 1D). Compared the average weight to the original, the severe colitis group weight was 90.65±10.16% (*p<*0.01). Meanwhile, the 20 mg/kg CK group was 95.45±7.63% and the BBR group was 94.94±8.39% of their original body weight at the average (Fig. 1D). Obviously, high concentrations of CK and BBR have significant effects on alleviating the DSS-induced colitis mice weight loss.

Moreover, shortening of the colon length is also a marker for colitis. Compared with control group and two colitis models, the colon length of the light colitis group was 3.30±0.53 cm shorter at the average (Fig. 1E, 1G, *p*<0.001) and the severe one was about 4.53±0.48 cm shorter at the average (Fig. 1F, 1G, *p*<0.001) either. Strikingly, CK and BBR exerted preventive effects on the colon lengh of both mild colitis mice and sever colitis mice.

**Figure 2:**
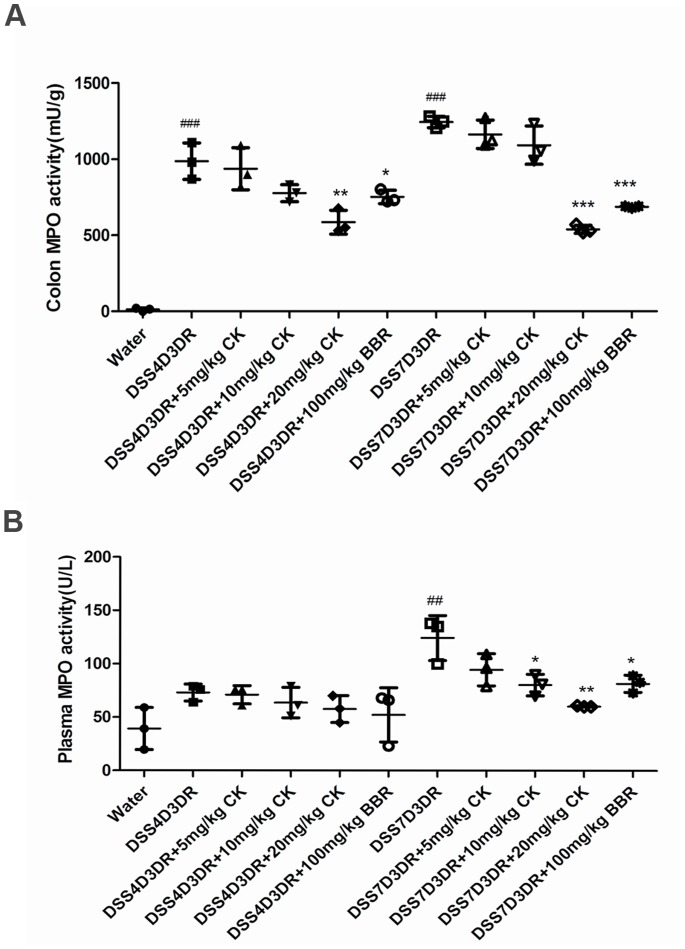
The MPO levels in colon tissues and mice plasma. (A) The levels of MPO in colon tissues. (B) The levels of MPO in mice plasma. ## *p<*0.01, ### *p<*0.01 compared with the control group. *p compared with DSS4D3DR or DSS7D3DR group. n = 3. *p<0.05, **p<0.01, ***p<0.001.

Furthermore, DSS induces neutrophil infiltration in the colon leading to increasing colonic MPO activity, which is treated as another inflammatory marker for colitis. In mild colitis mice groups, after 20 mg/kg CK and 100 mg/kg BBR treatment, MPO activity in the colon was reduced by 401±41.29 mU/g (*p<*0.01) and 235±76.37 mU/g (*p<*0.05) (Fig. 2A) compared to the model group. Meanwhile, 20 mg/kg CK and 100 mg/kg BBR treatment reduced the MPO by 705.3±11.27 mU/g (*p<*0.001) and 558.3±30.71 mU/g (*p<*0.001) in severe colitis mice compared to the model group, respectively (Fig. 2A). We also found that the reducing tendency of MPO after CK and BBR treatment in colitis mice plasma was the same as the colon MPO activity (Fig. 2B). Above results indicated that DSS-treated mice demonstrated colon injury and acute colitis (mild colitis and sever colitis), including body weight loss, colon length shorten and MPO activity, all of which could be reduced by CK and BBR.

### Histology of DSS-induced Colitis in Mice

**Figure 3:**
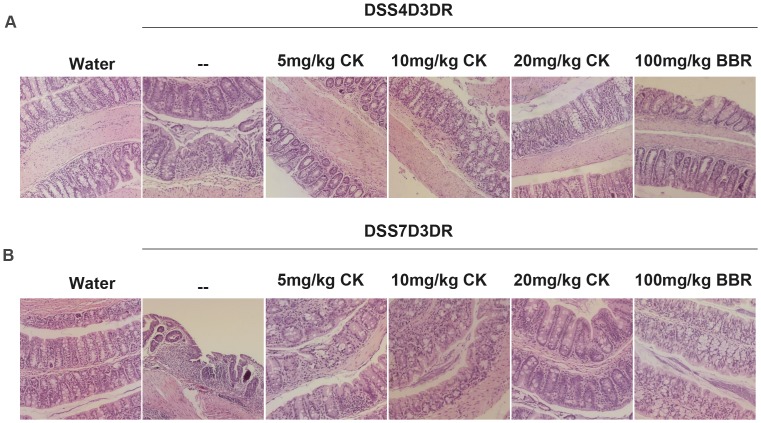
Histology analyze of DSS-induced colitis in mice. (A) Paraffin embedded colon sections were stained with hematoxylin and eosin for light microscopic assessment of epithelial damage of colitis mice, n = 3.

DSS-induced mice emerge massive colon ulceration, crypt damage, and severe inflammation [4], [5]. These abnormalities were reduced by treatment with BBR which we have authenticated in previous work [10]–[12]. In order to confirm more possible functional mechanisms of CK, we then did the H&E staining in paraffin-embedded colon tissues sections (n = 3, Fig. 3). DSS treatment caused no significant observable macroscopic changes in colonic tissue architecture between the experimental groups, including the untreated mice. However, DSS treatment tended to accelerate initial damage to the mucosa, characterized by the loss of goblet cells and the occurrence of a more diffuse crypt architecture compared to the colon of healthy mice in H&E staining sections (Fig. 3A, 3B). Additionally, the accumulation of neutrophils infiltrating the lamina propria could be observed more frequently in colonic slices of DSS-treated mice in both mild colitis mice and severe colitis groups (Fig. 3A, 3B). When treated with different concentrations of CK and BBR, the severity of inflammation seemed to be much lower (Fig. 3A, 3B). These data suggest that CK and BBR exerted therapeutic effects on both mild and severe colitis associated with intestinal epithelial cell injury repairmen, particularly the high concentration of CK.

### CK Reduced Pro-inflammatory Cytokines Production in DSS-treated Mice

**Figure 4:**
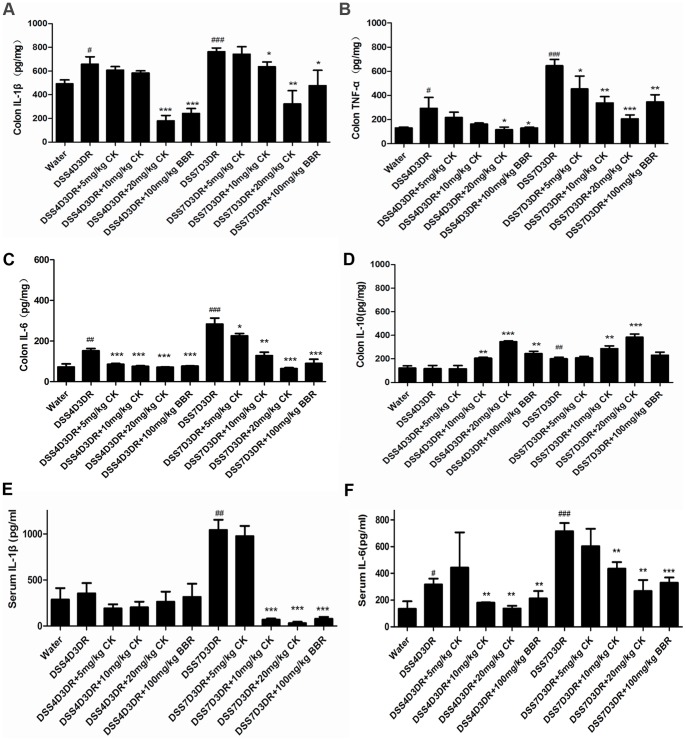
CK reduces production of inflammatory cytokines in DSS-induced colitis in mice. The colon tissues and mice serum were collected as the mean shows in method and materials. Before it performed, the tissues were fixed with ice-cold PBS in 1∶9 rations, homogenated, and centrifugalized. The supernatants were collected and IL-1β, TNF-α, IL-6 and IL-10 expressions were measured by ELISA (n = 3). The results were shown in (A), (B), (C) and (D), respectively. (E) and (F) The mice serum is detected the production of IL-1β, IL-6 directly by ELISA (n = 3). # *p<*0.05, ## *p<*0.01, ### *p<*0.01 compared with the control group. *p compared with DSS4D3DR or DSS7D3DR group. *p<0.05, **p<0.01, ***p<0.01.

The biological activities of CK include anti-metastatic, anti-carcinogenic, and anti-allergic effects, but its role in DSS-induced inflammatory signaling is poorly understood [20]–[24]. To examine the function of CK on treating colitis, the production of cytokines in colon tissues and serum were detected. In the mild colitis group, the expression of pro-inflammatory cytokines TNF-α, IL-6, IL-1β were increased to about 1.5 folds (Fig. 4A, 4B, 4C, *p<*0.05), and in the severe colitis group IL-6, TNF-α, IL-1β expression were increased to about 3, 2, and 1.5 folds (Fig. 4A, 4B, 4C) compared to the water group. After the treatment of CK, it was obvious that CK inhibited the DSS-induced expression ofpro-inflammatory cytokines and increased anti-inflammatory cytokine (IL-10) production (Fig. 4D). Investigation of the production of IL-1β and IL-6 in colitis mice serum confirmed our results. 7-day DSS treatment group increased the expression of IL-1β about 5 folds (*p<*0.01) compared to the water group (Fig. 4E) and then the 10 mg/kg, 20 mg/kg CK and 100 mg/kg BBR treatment groups decreased the IL-1β production all about 10 folds (*p<*0.001) (Fig. 4E). But the 4-day DSS treatment and 3-day recovery group didn’t have significant results. The mild colitis group increased the expression of IL-6 about 2 folds (Fig. 4F, *p<*0.05) and the severe group increased about 5 folds (Fig. 4F, *p<*0.01) compared to the water group. CK can also down-regulated the IL-6 secretion in mice serum. Obviously, the serum levels of IL-1β and IL-6 were significantly suppressed in mice after administration of CK (Fig. 4E, 4F). Together, our findings suggest that CK, similar to BBR, suppressed pro-inflammatory cytokines production in DSS-induced mild and severe colitis in both mice tissues and blood.

### CK Inhibited the NF-κB Pathway in DSS-induced Colitis in Mice

**Figure 5:**
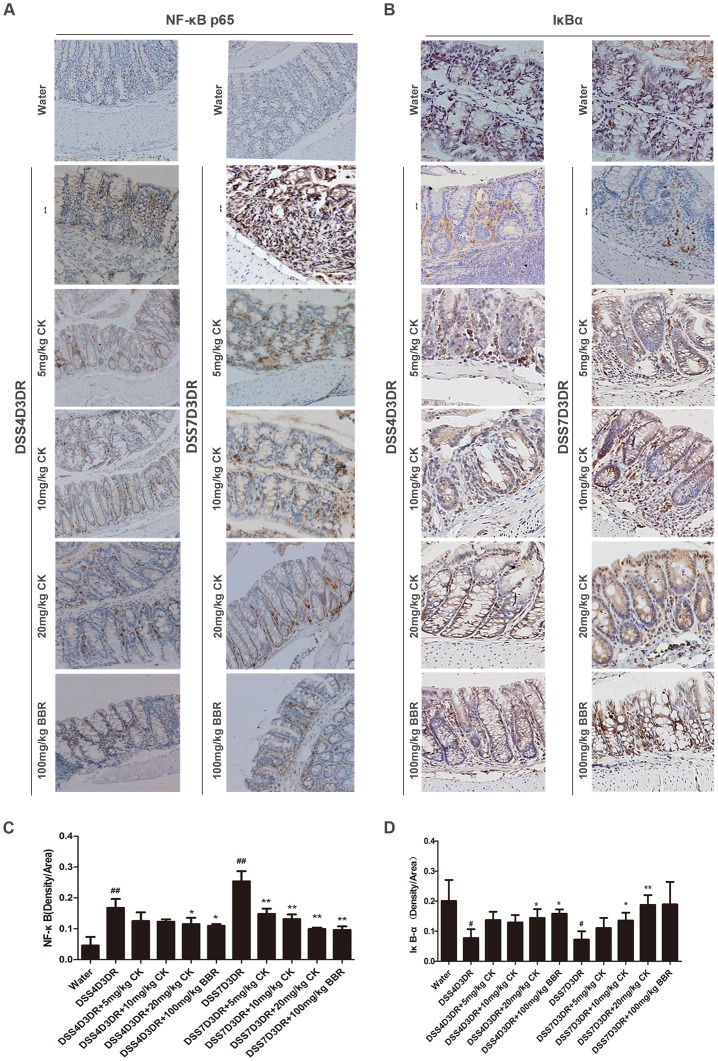
CK inhibits the NF-κB activation in DSS-induced colitis in mice. Representative images of IHC staining of NF-κB (p65) and IκB-α in mild colitis and severe colitis. (A) The expression of NF-κB (p65). (B) The expression of IκB-α. Software used 20 images. Three animals were analyzed for each type of mice. (C) Quantification of NF-κB (p65) positive density in each group. (D) Quantification of IκB-α positive density in each group (n = 3). # *p<*0.05, ## *p<*0.01 compared with the control group. *p compared with DSS4D3DR or DSS7D3DR group. *p<0.05, **p<0.01.

The transcription factor NF-κB is a central mediator in inflammation and several other cellular responses [12], [25]. NF-κB is translocated to the nucleus by phosphorylation of IκB and subsequent degradation of IκB subunit. BBR can inhibit the activation of the NF-κB pathway in DSS-induced mice [1], [26]. To reveal the anti-inflammatory mechanisms of CK on treating colitis in mice, IHC analysis showed that nuclear p65 was significantly reduced in CK and BBR treatment groups compared to control groups (Fig. 5A). Quantification of staining density in multiple samples indicated that in the 4-day DSS treatment group and 7-day water group, nuclear p65 increased 3 folds and 5 folds respectively, compared with the water group (Fig. 5C, *p*<0.01, n = 6). Yet, after administered CK and BBR for the following three recovery days, the nuclear p65 enormously decreased (Fig. 5C, n = 6). Nevertheless, 20 mg/kg CK on 4-day DSS treatment groups reduced the nuclear p65 by 2 folds (Fig. 5C, *p*<0.05, n = 6). We also examined the IκB-α expression in colon tissues and the results showed that IκB-α expression would be much higher in the CK and BBR treatment groups (Fig. 5B, n = 6). Obviously, the mild colitis groups and sever colitis groups presented the same tendency. Quantification of staining density of IκB-α, both in 4-day and 7-day DSS treatment groups, showed that CK and BBR have substantially facilitated the IκB-α expression (Fig. 5D, n = 6). Taken together, with the observation of nuclear p65 activation and IκB-α degradation, these results indicated that NF-κB signaling pathway was inhibited by CK and BBR.

**Figure 6:**
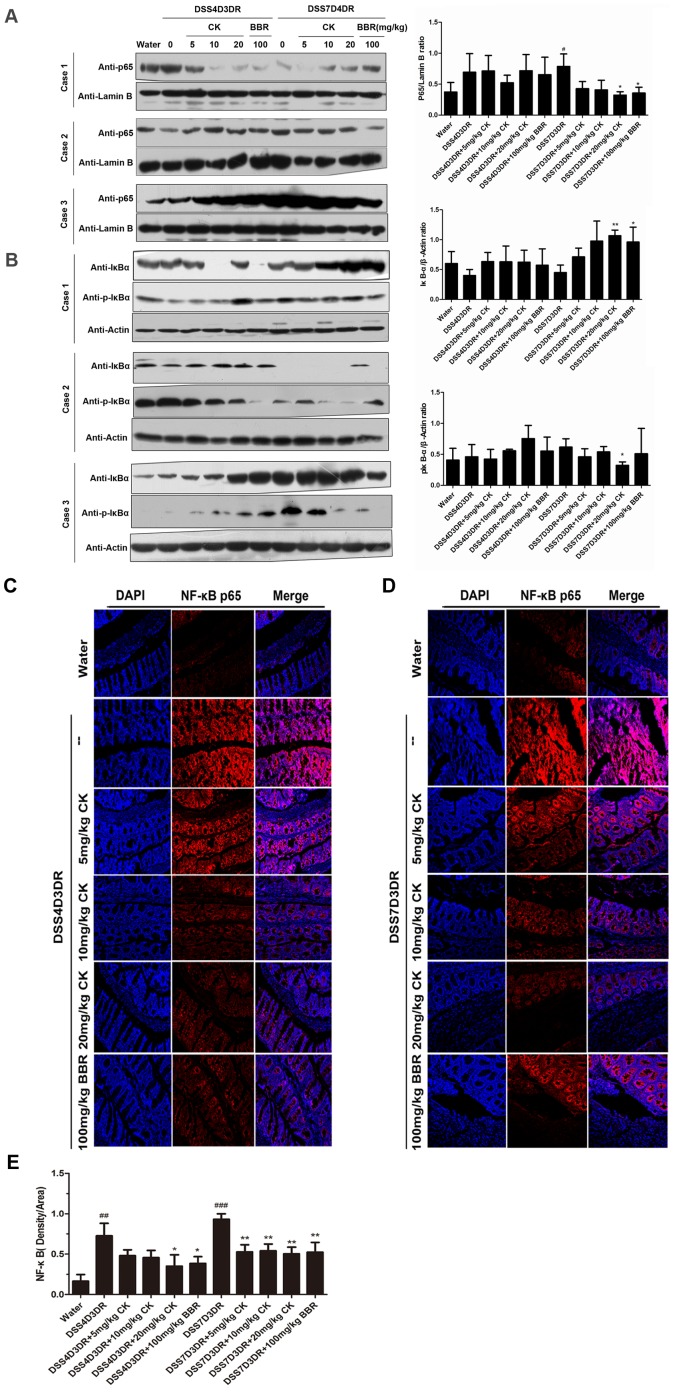
CK inhibits the NF-κB signaling activation in DSS-induced colitis mice. (A) Western blotting analysis of p65 expression in nucleus in different doses of CK and BBR mice group. Three animals were analyzed for each type of mice As shown in the left panel. In the right panel, the relative density of the activated nucleus p65 band was compared with the lamin B band in each group. (B) Analysis of IκB-α and p-IκBα in mild colitis and severe colitis mice tissues cytoplasm protein. In the right panel, the relative density of IκB-α and p-IκBα bands were compared with the β-actin band in each group. (C) and (D) Paraffin-embedded colon tissues sections of mild colitis group and severe colitis group were used to determine p65 translocated to the nucleus by immunofluorescence. (E) Quantification of NF-κB (p65) positive density in each group under 20X image. ## *p<*0.01, ### *p<*0.01 compared with the control group. *p compared with DSS4D3DR or DSS7D3DR group. *p*<0.05, **p<0.01.

Western blotting analysis was used to further confirm our results. Three colon tissues samples from each group and three separate tests were done. The expression of p65 was shown in figure 6A (left panel). The relative density of the activated nuclear p65 compared with Lamin B in each group was shown in figure 6A right panel. Strikingly, CK treatment caused a dose-dependent decrease in p65 expression in colon tissues. The 20 mg/kg CK group reduced the p65 expression by half (Fig. 6A, right panel, *p*<0.05, n = 3). And BBR could also inhibit p65 access to nucleus (Fig. 6A, right panel, *p*<0.05, n = 3). In addition, in the cytosol, the IκB-α expression increased and p-IκBα decreased after CK and BBR treatment in three independent experiments (Fig. 6A, left panel, n = 3). The relative density of IκB-α and p-IκBα compared with the β-actin in each group was shown in figure 6B right panel. Nevertheless, those results about the protein expression of NF-κB signaling pathway in the whole colon seem not very obvious. According to previous work, colitis signed by intestinal epithelial barrier function damage, and triggered innate immune cells such as macrophages and intestinal epithelia cells secreting cytokines and chemokines, which mostly leading to activate NF-κB pathway and then regulate the inflammatory responses. BBR reduced the colon injury via inhibiting the colon tissues NF-κB pathway activation. So, the notion that the anti-inflammation effects of CK may also regulate the colon tissue NF-κB pathway activation will be more acceptable. However, due to mice individual difference, more samples must be done to verify the statements better.

Paraffin-embedded colon tissues sections were used to determine p65 translocation to the nucleus by immunofluorescence and visualized by fluorescence microcopy (Fig. 6C, 6D). Quantification of fluorescence density was shown in figure 6E. Nucleus p65 expression distinctly reduced after CK and BBR treatment (Fig. 6C, 6D, 6E).

From above, we demonstrated for the first time that CK prevented the progression of DSS-induced colitis mice through inhibiting NF-κB pathway activation, thus leading to preventing p65 translocation to nucleus and IκB-α phosphorylation.

### CK Inhibited Activation of NF-κB Pathway in Macrophages

**Figure 7:**
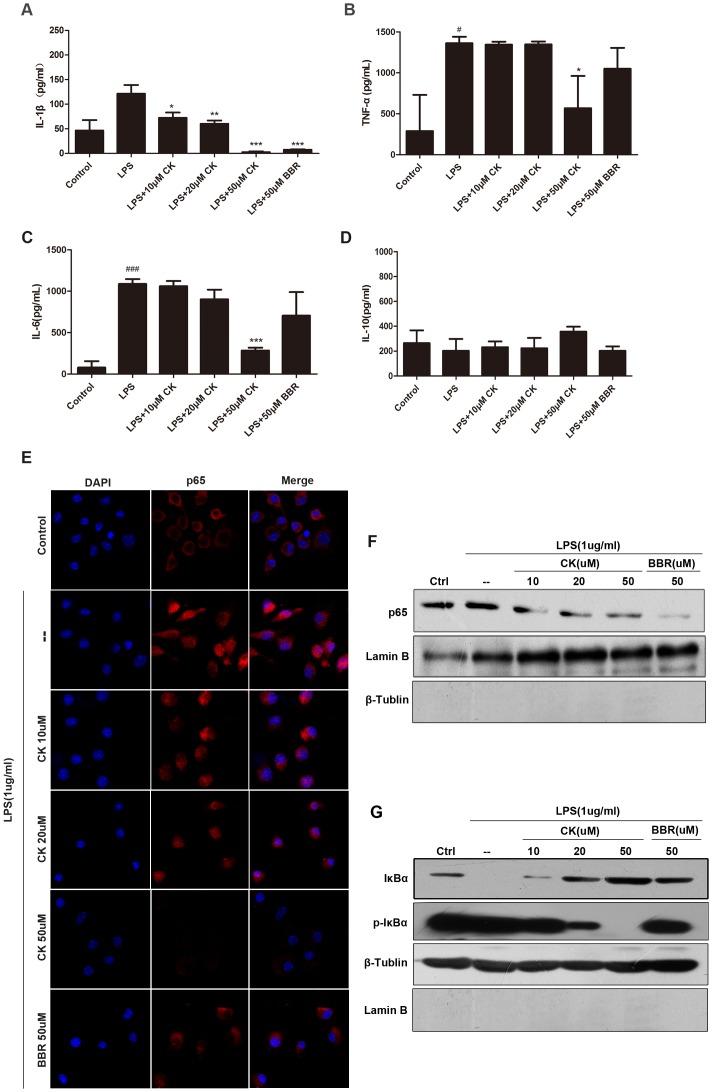
CK inhibits the NF-κB signaling activation in macrophage RAW 264.7 cells. The RAW 264.7 Cells were stimulated with lipopolysaccharide (LPS) (1 µg/ml) for 10 h in the presence or absence of different dose of CK (10 µM, 20 µM, 50 µM) and BBR (50 µM). The supernatants were subsequently harvested and measured by ELISA (n = 3). IL-1β, TNF-a, IL-6 and IL-10 expressions were shown in (A), (B), (C), and (D). (E) NF-κB nuclear translocation was detected by immunofluorescence analysis using an antibody for the p65. (F) Western blotting results were shown p65 expression in nuclear fractions. (G) IκB-α and p-IκBα expression in cytosolic fractions. All those have done three separate tests. # *p<*0.05, ### *p<*0.01 compared with the control group. *p compared with DSS4D3DR or DSS7D3DR group. *p<0.05, **p<0.01, ***p<0.01.

CK and BBR have demonstrated potent anti-inflammation effects in *vivo.* It has been reported that in peritoneal macrophages and RAW264.7 cell lines, BBR can down-regulate NF-κB pathway that mediate pro-inflammatory cytokines production, and contribute to relieve the colitis injury, which suggests that BBR has influence on regulating the innate immune responses. CK, as the active metabolite responsible for the anti-inflammatory effect of ginsenoside, prompted us to investigate its functional mechanismon LPS-induced inflammation *in vitro*. LPS is one of the most potent pro-inflammatory agonists for monocytes and macrophages, and macrophage is one of the functional innate immune cells in inflammatory responses and it regulates innate immune responses by providing cytokine [27]–[29]. Sustained stimulation with LPS could active NF-κB pathway in RAW 264.7 cells [30]. In this work, RAW 264.7 cells were treated with lipopolysaccharide (LPS) (1 µg/ml) for 10 h in the presence or absence of different dose of CK (10 µM, 20 µM, 50 µM) and BBR (50 µM). The control (cell-only) group was used as a positive control for anti-inflammatory activities. In the LPS exposure group, IL-1β, IL-6, TNF-a productions increased dramatically to 5, 10, 8 folds respectively, compared to the control group. However, CK reduced IL-1β, IL-6 and TNF-a production in a dose-dependent manner (Fig. 7A, 7B, 7C). Nevertheless, IL-10 production changed a little (Fig. 7D). BBR (50 µM) also have influence on reducing the pro-inflammation production, expect for IL-10 (Fig. 7A, 7B, 7C, 7D), significantly. In summary, CK and BBR have significant effects on reducing pro-inflammatory cytokines production *in vitro*, and regulating the inflammatory responses in *vivo* via impacting the production of cytokines, which were provided by immune cells, such as macrophages.

According to the previous work, immunofluorescence and western blotting were used to explore the inhibitory effects of CK on inflammation (Fig. 7E, 7F, 7G). The location of nucleus p65 was detected by fluorescence microcopy in RAW 264.7 cells treated with lipopolysaccharide (LPS) (1 µg/ml) for 10 h in the presence or absence of different doses of CK (10, 20, and 50 µM) and BBR (50 µM) It was obvious that CK prevented p65 translocation to nucleus in a concentration-dependent manner. Moreover, western blotting results indicated that CK promoted IκB phosphorylation in a concentration-dependent manner.

Taken together, these data suggest that CK and BBR have influence on reducing LPS-stimulated pro-inflammatory cytokines, increasing anti-inflammatory cytokine expression, contributing to inhibition of the activation of NF-κB pathway, restraining p65 translocated to nucleus, and inhibiting phosphorylation of IκB of macrophage cells, which may serve as mechanism for CK down-regulated inflammatory responses of intestinal disorder *in vivo*.

## Discussion

The evaluation of ancient herbal products that have been widely used for many diseases, such as treating diarrhea and bacterial and parasitic infection, may provide insight into developing these herbs into new therapies for other inflammation treatment [1], [31]. CK is an intestinal bacterial metabolite of panaxoside, which have been widely reported to exert anti-inflammation and anti-cancer activity [20]–[26]. *Our studies* and *other previous* work have demonstrated the anti-growth effect of CK in different kinds of tumors cells, such as hepatocellular carcinoma [13]. Although, ginsenoside Rb1 and its metabolites have been confirmed playing an beneficial role in inflammatory responses in TNBS-induced colitis by inhibiting IRAK-1 activation, which contributes to activate IKKβ, linking with NF-κB and MAPK pathways [12]. BBR has been used to treat bacteria associated diarrhea, intestinal parasitic infections, and ocular trachoma infections for several decades [27]. We have proved that BBR induces death in colon tumor cells. Recent work has indicated that BBR promotes recovery of colitis and ameliorate inflammatory responses in DSS-treated mice by promoting colon peritoneal macrophages apoptosis, and decreasing pro-inflammatory production in colonic macrophages and macrophages cell lines [1]. However, the functional mechanisms of CK and BBR on treating those diseases are not clearly known. The purpose of this work was to determine the effects of CK on treatment of the progression of colitis and to provide insight into the potentially functional mechanism.

In the present study, BBR served as a positive agent for treating the DSS-induced colitis mice mode. We showed that CK exerts inhibitory inflammatory effects not only on mild colitis (4-day DSS-induced colitis), but also on severe colitis (7-day DSS-induced colitis), including protection of the weight loss and colon tissues injury, and preservation of of colon length. Furthermore, similar as BBR, CK treatment reduced MPO activity in mice colon and mice plasma in dose-dependent manner. We observed that CK and BBR reduced the production of the pro-inflammatory cytokines, such as IL-1β, IL-6 and TNF-α, but not the anti-inflammatory cytokine, such as IL-10 in colon tissue. In addition, similar results were found in colitis mice serum. *In vitro*, LPS-stimulated macrophage RAW 264.7 cells were treated with BBR and CK and then the supernates cytokines generation was measured. Those results show CK, same as BBR, protected the mice from the colitis injury by increasing the production of the anti-inflammatory cytokines and reducing the pro-inflammatory cytokines in concentration-dependent manners. Based on the cytokines levels that are regulated by CK and BBR, it was possible that they ameliorated colitis through regulating the innate immune system.

As we all known, production of cytokines and chemokines by immune/inflammatory cells that activate transcription factors such as NF-κB in pre-malignant cells to induce genes that stimulate cell proliferation,survival, and growth, as well as angiogenesis, and invasiveness motility [32]–[35]. In details, NF-κB (p65) is located in cytoplasm and bounds to IκB as an inactive complex [32], [33]. The phosphorylation and subsequent degradation of IκB result in separation of the complex, and then NF-κB is activated. The activated NF-κB migrates into the nucleus, and causes the expression of inflammatory cytokines, such as TNF-a, IL-6 and IL-8 [35]. Meanwhile, NF-κB can be activated by inflammatory factors such as IL-1β and TNF-α [35], [36]. TNF-α can activate macrophages and initiate immune responses by stimulating secretion of other cytokines [37], [38]. IL-1β, mainly synthesized by macrophages, is also an inflammatory cytokine which play important roles in the acute phase response [39], [40]. In addition, NF-κB signaling pathway is the downstream pathway of LPS-mediated transduction pathways [29]. As it is been well accepted, in difference forms of inflammation, innate immune cells (including macrophages, neutrophils, mast cells, etc) and adaptive immune cells (T and B lymphocytes) are connected with each other by means of direct contact of cytokines and chemokines, leading to regulating the innate immune responses [40]. Unrestrained reactions may exaggerate inflammatory response and lead to continuous events of injury and tissues damage [41], [42].

**Figure 8:**
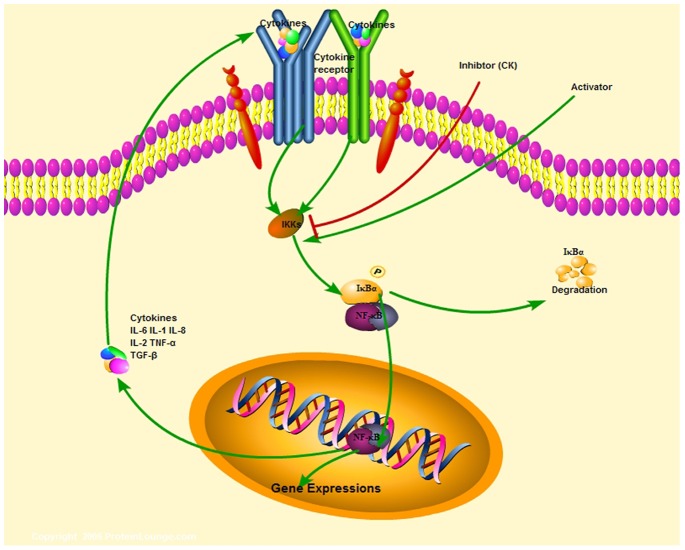
CK prevents inflammatory responses *via* inhibiting NF-κB signaling pathway activation. CK exerts therapy effects on the progression of DSS-induced colitis in mice, and the potential mechanisms may contribute to regulating the innate immune cells such as macrophages activities through modulating NF-κB signaling pathways activation.

Our data showed that CK and BBR down-regulated colonic tissues NF-κB signaling pathways in response to the recovery of 4 days DSS-induced and 7 days DSS-induced colitis *in vivo* and inhibited the activation of macrophages NF-κB signaling pathways in LPS-stimulated inflammation *in vitro*. In addition, our data suggest that CK and BBR mediate the progression of the colon disease by regulating innate immune cells, linked with the production of cytokines and chemokines. The potentially singling pathway of this work was shown in figure 8.

In summary, our data indicated that CK exerts effient therapy effects on DSS-induced progression of colitis in mice, and the potential mechanisms for regulating this intestinal inflammation may contribute to regulating the innate immune cells such as macrophages activities through modulating of NF-κB signaling pathways activation that dominate the production of cytokines and chemokines. Since CK has been widely used for treating with many diseases without significant side effects on patients, it has the potential to be developed into a drug for intestinal inflammatory diseases.
