# Expression of Concern: Ginsenoside Metabolite Compound K Promotes Recovery of Dextran Sulfate Sodium-Induced Colitis and Inhibits Inflammatory Responses by Suppressing NF-κB Activation

**DOI:** 10.1371/journal.pone.0338671

**Published:** 2025-12-11

**Authors:** The PLOS One Editors

After publication of this article [1], concerns were raised about results presented in Figs 3 and 7. Specifically:

- The ‘-‘panel of Fig 3A appears to partially overlap with the 20 mg/kg panel of Fig 3B.
- The β-Tubulin panel in Fig 7F appears similar to the Lamin B panel in Fig 7G.

The corresponding author stated that the 20 mg/kg CK panel in Fig 3B is incorrect, and provided a revised version of Fig 3 including the correct image from the original study along with the underlying images for Fig 3 in S1 File.

The corresponding author stated that the β-Tubulin panel in Fig 7F and the Lamin B panel in Fig 7G were duplicated in error and that it is not known which one is incorrect as the underlying images for these figures are no longer available. They provided data in support of the results reported in Figs 7F and 7G from later repeat experiments which had two independent replicates; the repeat western blot image files and densitometry data are provided in S2-S4 Files.

Members of the *PLOS One* Editorial Board reviewed S1-S4 Files and stated that the updated 20 mg/kg CK panel in Fig 3B and the repeat data for Figs 7F and G appear to accurately support the conclusion that BB and CX inhibit LPS-induced NF-κB, but noted the limitation of a small sample size for the repeat western blot experiment.

In addition, there is an error in the last sentence of the second paragraph of the Results subsection titled CK Inhibited Activation of NF-κB Pathway in Macrophages. The correct sentence is: Moreover, western blotting results indicated that CK inhibited IκB phosphorylation in a concentration-dependent manner.

During editorial follow-up, members of the *PLOS One* Editorial Board noted that the study design of [1] does not test causality as analysis in cells or organisms with targeted deletions in the pathway needed to abrogate the effect are not included in [1], and, as such, statements regarding causal relationships between NF-κB inhibition and the beneficial effects of compound K on DSS-induced colitis are speculative. Examples of statements that are unsupported by the study design include ‘by Suppressing NF-κB Activation’ in the title, abstract, and introduction of [1], “through inhibiting NF-κB pathway activation, thus leading to...” in the Results section, and “through modulating NF-κB signalling pathways activation that dominate the production of cytokines” in the Discussion section. Members of the *PLOS One* Editorial Board stated that while the data demonstrate that CK treatment is associated with both improved colitis outcomes and suppression of NF-κB activation, they do not include mechanistic experiments that would directly establish causality. PLOS also has concerns about the small sample sizes reported for the *in vivo* experiments.

The corresponding author stated that the individual-level underlying data and statistical analysis for Figs 1-2 and 4-7 are no longer available.

The *PLOS One* Editors issue this Expression of Concern to inform readers of the above issues, which call into question the reliability of the article’s conclusions.

**Fig 3:**
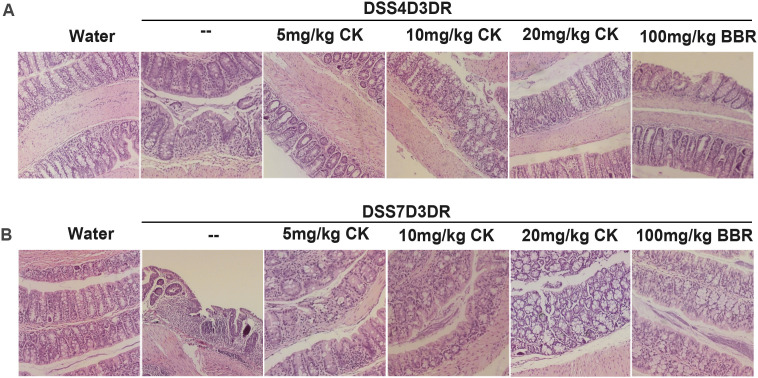
Histology analyze of DSS-induced colitis in mice. **(A)** Paraffin embedded colon sections were stained with hematoxylin and eosin for light microscopic assessment of epithelial damage of colitis mice, n = 3.

## Supporting information

S1 File Underlying images supporting Fig 3. (RAR)

S2 File Raw, uncropped blots from the replication experiments supporting the results reported in Figs 7F and 7G of [1]. Boxed areas indicate the cropped regions displayed in the respective figures. Cytosolic and nuclear proteins were isolated after cells treated with LPS, CK or BBR. Then western blotting was performed with cytoplasmic samples on the left and nuclear samples on the right of the gel. After blocking, membranes were incubated overnight at 4°C with the primary antibody in blocking buffer using p-65, β-tubulin plus Lamin B, IκBα or p-IκBα antibody, respectively. Then, membranes were washed and incubated with an HRP-conjugated secondary antibody. At last, the uncropped membranes were exposure. Cytoplasmic samples were loaded on the left and the nuclear samples on the right of the gel. For the 2nd and 5th blots, β-tubulin and Lamin B antibodies were incubated together.
(JPG)

S3 File Raw, uncropped blots from the second replicate experiment supporting the results reported in Figs 7F and 7G of [1]. (JPG)

S4 File Densitometric analysis of replication experiments in support of Fig 7. (XLSX)
