# Supplementary figures and images for: Expression of Concern: Ginsenoside Metabolite Compound K Promotes Recovery of Dextran Sulfate Sodium-Induced Colitis and Inhibits Inflammatory Responses by Suppressing NF-κB Activation
Source: PLoS One. 2025 Dec 11;20(12):e0338671. doi: 10.1371/journal.pone.0338671 (PMC12698003; doi:10.1371/journal.pone.0338671)

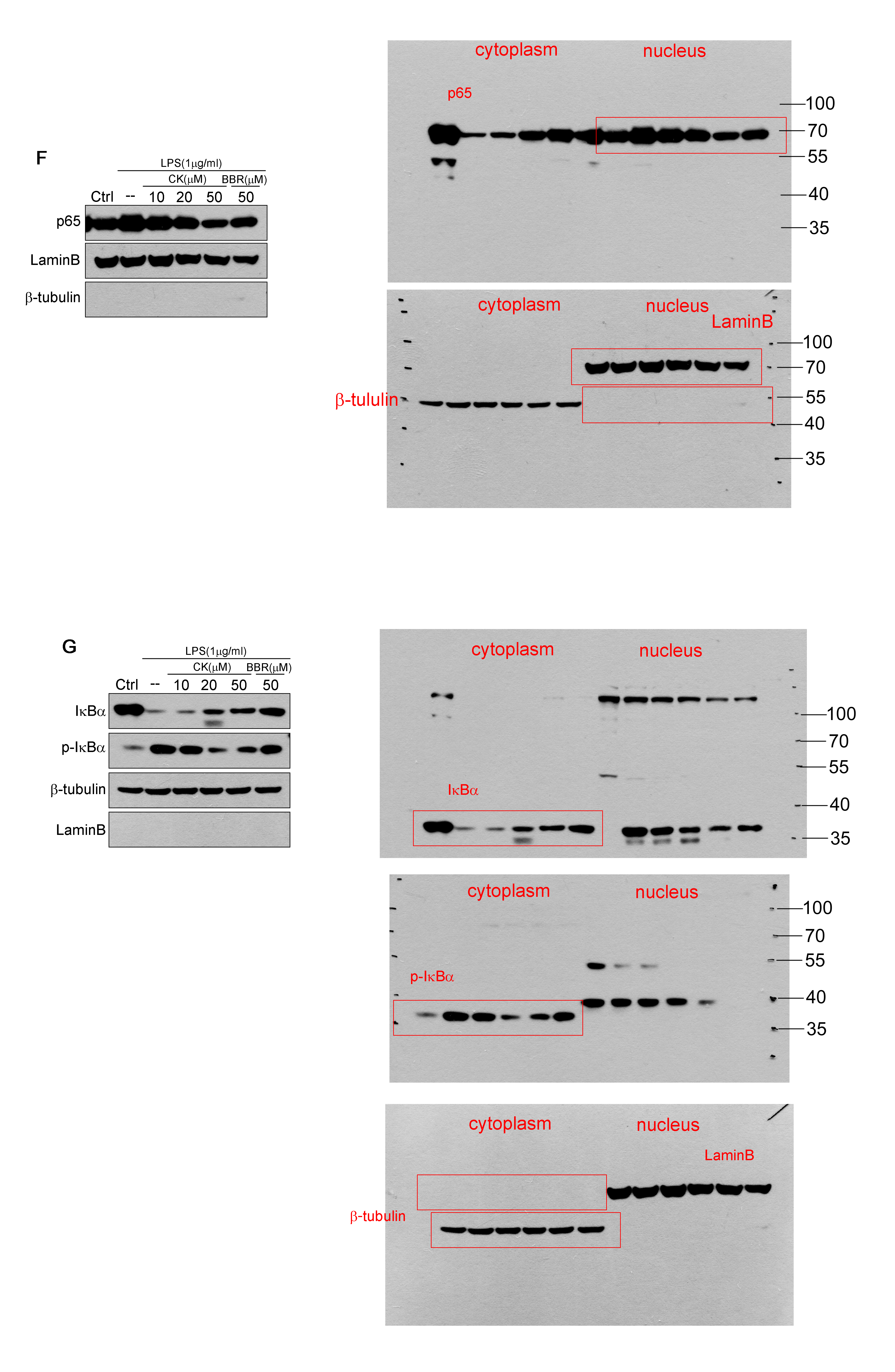

Supplement: S2 File — Boxed areas indicate the cropped regions displayed in the respective figures. Cytosolic and nuclear proteins were isolated after cells treated with LPS, CK or BBR. Then western blotting was performed with cytoplasmic samples on the left and nuclear samples on the right of the gel. After blocking, membranes were incubated overnight at 4°C with the primary antibody in blocking buffer using p-65, β-tubulin plus Lamin B, IκBα or p-IκBα antibody, respectively. Then, membranes were washed and incubated with an HRP-conjugated secondary antibody. At last, the uncropped membranes were exposure. Cytoplasmic samples were loaded on the left and the nuclear samples on the right of the gel. For the 2nd and 5th blots, β-tubulin and Lamin B antibodies were incubated together. (JPG) [file pone.0338671.s002.jpg]

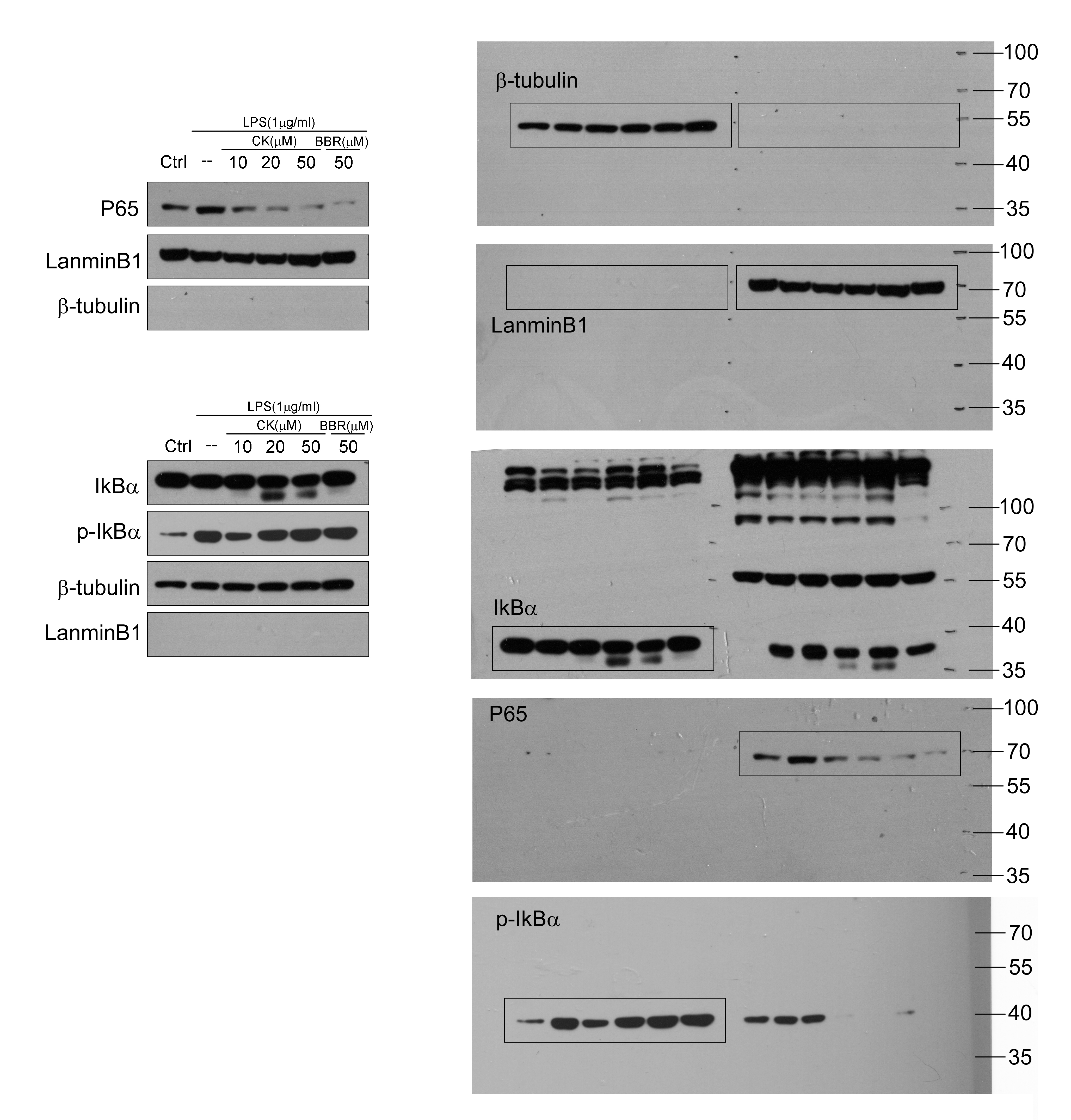

Supplement: S3 File — (JPG) [file pone.0338671.s003.jpg]
